# Dysregulation of TCONS_00006091 contributes to the elevated risk of oral squamous cell carcinoma by upregulating SNAI1, IRS and HMGA2

**DOI:** 10.1038/s41598-024-60310-4

**Published:** 2024-04-26

**Authors:** Danhua Ma, Jijun Chen, Yuyuan Shi, Hongyan Gao, Zhen Wei, Jiayan Fan, Liang Wang

**Affiliations:** Department of Stomatology, Ningbo No. 2 Hospital, No. 41 Northwest Street, Ningbo, 315010 Zhejiang China

**Keywords:** lncRNA, Oral squamous cellular carcinoma, HMGA2, IRS, SNAI1, Proliferation, Oral cancer, Tumour biomarkers

## Abstract

In this study, we aimed to study the role of TCONS_00006091 in the pathogenesis of oral squamous cellular carcinoma (OSCC) transformed from oral lichen planus (OLP). This study recruited 108 OSCC patients which transformed from OLP as the OSCC group and 102 OLP patients with no sign of OSCC as the Control group. ROC curves were plotted to measure the diagnostic values of TCONS_00006091, miR-153, miR-370 and let-7g, and the changes in gene expressions were measured by RT-qPCR. Sequence analysis and luciferase assays were performed to analyze the molecular relationships among these genes. Cell proliferation and apoptosis were observed via MTT and FCM. TCONS_00006091 exhibited a better diagnosis value for OSCC transformed from OLP. OSCC group showed increased TCONS_00006091 expression and decreased expressions of miR-153, miR-370 and let-7g. The levels of SNAI1, IRS and HMGA2 was all significantly increased in OSCC patients. And TCONS_00006091 was found to sponge miR-153, miR-370 and let-7g, while these miRNAs were respectively found to targe SNAI1, IRS and HMGA2. The elevated TCONS_00006091 suppressed the expressions of miR-153, miR-370 and let-7g, leading to the increased expression of SNAI1, IRS and HMGA2. Also, promoted cell proliferation and suppressed apoptosis were observed upon the over-expression of TCONS_00006091. This study demonstrated that the expressions of miR-153, miR-370 and let-7g were down-regulated by the highly expressed TCONS_00006091 in OSCC patients, which accordingly up-regulated the expressions of SNAI1, IRS and HMGA2, resulting in the promoted cell proliferation and suppressed cell apoptosis.

## Introduction

Oral squamous cell carcinoma (OSCC) is a type of cancer which develops in the oral cavity and maxillofacial region. It is the most prevalent oral malignancy, accounting for approximately 80% of all malignant neoplasms of the oral cavity and maxillofacial region^1^. Although the incidence of oral cancer varies across the world, As it is widely recognized that the oral cavity ranks between the 6th to the 9th most common site for cancer^2^. Several factors contribute to OSCC, including smoking and drinking habit, exposure to ultraviolet radiation, malnutrition, genetic predisposition, or even human papillomavirus (HPV) infection^3,4^. The 5-year survival rate of OSCC, as reported previously, ranges significantly between OSCC patients without recurrence (92%) and OSCC patients with recurrence (30%)^5^. To promote the survival of OSCC, it is crucial to performed early detections such as regular dental check-ups and self-examination of the mouth^6^.

Long non-coding RNA (lncRNA) belongs to a type of RNAs of > 200 nt in length with no protein coding abilities^7^. It was estimated that less than 2% of the genome in human can be transcribed into mRNAs, while most of the genome is transcribed to non-coding RNAs^7^. lncRNAs participate in vital processes in the transcriptional, post-transcriptional as well as epigenetic regulation of gene expression to maintain normal cell and tissue growth as well as cell differentiation, along with other biological processes such as immune reactions as well as metabolic processes^8,9^.

An elevation in the expression of miR-145 as well as the associated down-regulated IRS-1 expression was shown to suppress the growth of colon cancer cells^10^. In addition, IRS-1 expression was discovered to be reduced in OSCC cells infected by HPV^11^. The negative regulatory relationship between the expression of IRS-1 and miR-370 seems to support the hypothesis suggesting that miR-370 targets the expression of IRS-1 in OSCC cells^12^.

As a key regulator of epithelial-mesenchymal transition, SNAI1 has been reported to be crucial for the progression of cancer, in respect to its functions such as enabling resistance to anti-cancer drugs or facilitating invasion by cancer cells^13^. In a previous study which investigated the expression of SNAI1 in oral cancer cells, SNAI1 was found to be up-regulated by the highly expressed transforming growth factor (TGF)-β in OSCC cells^14^. Moreover, with the presence of TGF-β, SNAI1 siRNA can mitigate malignant phenotypes of oral cancer^14^. Also, in another study which investigated the role of SNAI1 in areca quid chewing-associated OSCC, the elevated SNAI1 levels can mediate tumor differentiation and lymph node metastasis, while the up-regulation of SNAI1 was found to be a possible result of reactive oxygen species^15^.

The protein family of insulin receptor substrates (IRSs) includes six member proteins, namely IRS-1 to IRS-6, whose expression is elevated in cells with activated surface receptors such as insulin-like growth factor receptor 1 (IGF1R) as well as insulin receptor (IR)^16^. The phosphorylation of IRS mediated via the IGF axis is essential for recruiting PI3K in the activation of the AKT/mTOR pathway^17^.

The HMGA2 gene belongs to the gene family of high mobility group A (HMGA) proteins, which contain 4 members, i.e., HMGA1c, HMGA1b, HMGA1a, as well as HMGA2^18^. Upon the loss of inhibitory effect of HMGA2 on let-7, neoplastic transformation becomes more likely^19^. Previous research reported the significant role of HMGA2 in the metastasis of cancer via the regulation of EMT mediated through transcription factors including Slug, SNAI1, as well as Twist^20^. Previously, it was shown that high expression of HMGA2 is actually involved in the poor prognosis as well as distant metastasis of colon cancer^21,22^. Although some articles also showed the association between poor prognosis and HMGA2 overexpression in oral cancer, there were few studies which reported the high expression of HMGA2 in metastatic OSCC^23,24^.

Several miRNAs, including miR-153, miR-370 and let-7g, have been demonstrated to be involved in the pathogenesis of OSCC transformed from oral lichen planus (OLP)^25^. Accordingly, HMGA2 is implicated in the regulation of angiogenesis and may play a role as a biomarker in the distant metastasis and prognosis in malignant OSCC^23^. IRS has been reported to participate in the molecular mechanisms underlying oral tumorigenesis^12^. Also, a miR-153-3p/SNAI1 axis was reported in OSCC cells^26^. In this study, we aimed to study the role of TCONS_00006091 in the pathogenesis of OSCC transformed from OLP. By characterizing OSCC transformed from OLP, we aim to establish potential biomarkers for its early diagnosis.

## Materials and methods

### Patient recruitment

In this study, we recruited 108 patients with OSCC transformed from OLP as the OSCC group, and 102 OLP patients with a stable diagnosis of OLP without any sign of OSCC/malignant transformation as the Control group. The recruited patients must have complete clinical records, which include details about the duration and severity of OLP, treatments received, and any other relevant medical history, and diagnosed with OSCC who have a documented history of OLP were also eligible. Meanwhile, patients with concurrent oral diseases or conditions were excluded. Those with a history of other types of cancer, as well as patients who are immunosuppressed due to organ transplantation, chronic diseases, or medications, were excluded. Patients who received radiotherapy and/or chemotherapy before surgical sampling were excluded. Additionally, patients with severe systemic diseases that could affect survival or complicate treatment were excluded. The demographic and clinical characteristics of the two groups were collected and compared using the Student’s t tests. In addition, at the time when the patients were diagnosed of OSCC (OSCC group) or OLP (Control group), the peripheral blood samples and lesion tissue samples, along with their corresponding tumor-adjacent normal tissues, were collected from each subject in the two groups for the lncRNA microarray analyses to identify lncRNAs that show the most significant changes in their expression levels in terms of fold changes (the threshold for determining the lncRNAs showing the most significant changes was set to > twofold).

### Cell culture and transfection

Two human oral squamous carcinoma cell lines, HSC-3 cells and SCC-9 cells (ATCC, Manassas, VA, US), were cultured under conditions in strict accordance with the standard protocol recommended by the manufacturer on the product manual. In brief, the cells were maintained in the Dulbecco's Modified Eagle's Medium (Gibco, Thermo Fisher Scientific, Waltham, MA) supplemented with 100 ug/ml of streptomycin (Gibco, Thermo Fisher Scientific, Waltham, MA), 100 U/ml of penicillin (Sigma Aldrich, St. Louis, MO), 4 g/l of glucose, as well as 10% of fetal bovine serum (Gibco, Thermo Fisher Scientific, Waltham, MA). And the culture conditions were 37 °C, 5% CO2 and saturated humidity. The subculture of cells was carried out once every 4 days after the cells became over 90% confluence. During the transfection experiments, when the cells reached over 70% confluence, the cells were seeded into a 24-well tissue culture plate at a density of 1 × 10^5^ cells/well and cultured overnight. On the next morning, the cells were randomly divided into different cell models. In cell model 1, both the HSC-3 cells and the SCC-9 cells were randomly divided into 2 groups, a NC group (HSC-3 and SCC-9 cells transfected with a negative control empty plasmid) and a p-TCONS_00006091 group (HSC-3 and SCC-9 cells transfected with the plasmids carrying TCONS_00006091). In cell model 2, both the HSC-3 cells and the SCC-9 cells were also randomly divided into 2 groups, a NC shRNA group (HSC-3 and SCC-9 cells transfected with the plasmids carrying a scramble negative control shRNA) and a p-TCONS_00006091 shRNA group (HSC-3 and SCC-9 cells transfected with the plasmids carrying TCONS_00006091 shRNA). The transfection was carried out by using Lipofectamine 2000 (Invitrogen, Carlsbad, CA) in strict accordance with the standard protocol recommended by the transfection reagent manufacturer on the product manual. At 48 h after the transfection, the cells were harvested for the subsequent analysis of target gene expression.

### RNA isolation and real-time PCR

For collected tissue samples, the total RNA extraction was conducted through 2 steps: In the first step, the tissue samples were homogenized by using a Precelyss 24 homogenization machine (Precelyss, Bertin, France) equipped with a CKMix ceramic-bead probe (Ozyme, Paris, France). In the second step, 5% (w/v) of the lysis buffer in a mirVana microRNA isolation assay kit (Ambion, Austin, TX) was added to each homogenized sample in strict accordance with the standard protocol recommended by the assay kit manufacturer on the product manual. For collected cell samples, the total RNA extraction was conducted directed by adding 5% (w/v) of the lysis buffer in the mirVana microRNA isolation assay kit (Ambion, Austin, TX) to each homogenized sample and following operations carried out in strict accordance with the standard protocol recommended by the assay kit manufacturer on the product manual. The integrity as well as concentration of total RNA extracted from each sample was analyzed by utilizing a BioAnalyzer 2100 (Agilent Technologies, Mountain View, CA) in strict accordance with the standard protocol recommended by the instrument manufacturer on the product manual. In the next step, different approaches were adopted for the mRNA analysis and miRNA analysis, respectively. For the miRNA analysis, the samples of extracted total RNA were converted into cDNA by making use of a NCode VILO miRNA cDNA synthesis assay kit (Invitrogen, Carlsbad, CA) in strict accordance with the standard protocol recommended by the assay kit manufacturer on the product manual. For the mRNA analysis, the samples of extracted total RNA were converted into cDNA via reverse transcription by making use of a SuperScript II Reverse Transcription assay kit (Invitrogen, Carlsbad, CA) in strict accordance with the standard protocol recommended by the assay kit manufacturer on the product manual. Finally, the quantitative real time PCR was carried out on a PRISM 7900 quantitative real time PCR machine (ABI, Foster City, CA) by making use of a QuantiFast SYBR Green master mix assay kit (Qiagen, Germantown, MD) in strict accordance with the standard protocol recommended by the assay kit manufacturer on the product manual. Based on the analysis of the melting curves, the relative expression levels of TCONS_00006091, miR-153, miR-370, let-7g, SNAI1, IRS, and HMGA2 in each sample were calculated by making use of the 2^−ΔΔCt^ technique. The sequences of the primers used in this study are: miR-153 forward: 5′-TTGCATAGTCACAAAAGTG-3′; miR-153 reverse: 5′-GAACATGTCTGCGTATCTC-3′; miR-370 forward: 5′-TGCTGGGGTGGAACCT-3′; miR-370 reverse: 5′-GAACATGTCTGCGTATCTC-3′; let-7g forward: 5′-TGAGGTAGTAGTTTGTACA-3′; let-7g reverse: 5′-GAACATGTCTGCGTATCTC-3′; SNAI1 forward: 5′-TGCCCTCAAGATGCACATCCGA-3′; SNAI1 reverse: 5′-GGGACAGGAGAAGGGCTTCTC-3′; IRS forward: 5′-GCGCAGGCACCATCTCAACAACC-3′; IRS reverse: 5′-GCACGCACCCGGAAGGAACC-3′; HMGA2 forward: 5′-GAAGCCACTGGAGAAAAACGGC-3′; HMGA2 reverse: 5′-GGCAGACTCTTGTGAGGATGTC-3′.

### Western blot analysis

The collected samples were first lysed in a RIPA buffer (Invitrogen, Carlsbad, CA) in strict accordance with the standard protocol recommended by the manufacturer on the product manual. Then, the sample proteins were resolved by 10% SDS-PAGE and blotted onto a NC membrane, which was blocked with 5% skim milk and incubated sequentially with primary anti-SNAI1, anti-IRS, and anti-HMGA2 antibodies as well as HRP-tagged secondary antibodies (Abcam, Cambridge, MA) in strict accordance with the incubation conditions recommended by the antibody manufacturer on the product manual. Finally, the color of protein bands was developed by making use of an ECL reagent (Invitrogen, Carlsbad, CA) in strict accordance with the standard protocol recommended by the manufacturer on the product manual and the relative expression of SNAI1, IRS, and HMGA2 proteins in each sample was calculated by using the Volume One software (Bio-Rad laboratories, Hercules, CA).

### Vector construction, mutagenesis, and luciferase assay

In our preliminary bioinformatic analysis, the results predicted the presence of a potential miR-153 binding site on TCONS_00006091, the presence of a potential miR-370 binding site on TCONS_00006091, the presence of a potential let-7g binding site on TCONS_00006091, the presence of a potential miR-153 binding site on SNAI1 mRNA, the presence of a potential miR-370 binding site on IRS mRNA, and the presence of a potential let-7g binding site on HMGA2 mRNA. Accordingly, we carried out luciferase assays in HSC-3 and SCC-9 cells to validate the above predictions regarding the potential relationships between TCONS_00006091 and miR-153, between TCONS_00006091 and miR-370, between TCONS_00006091 and let-7g, between miR-153 and SNAI1 mRNA, between miR-370 and IRS mRNA, and between let-7g and HMGA2. In brief, the wild type sequence of TCONS_00006091 containing the binding sites of miR-153, miR-370 and let-7g was cloned into pcDNA luciferase vectors (Promega, Madison, MI) downstream of the luciferase report gene to generate wild type plasmids of TCONS_00006091. At the same time, site directed mutagenesis was carried out by using a Quick Change mutagenesis assay kit (Stratagene, San Diego, CA) in strict accordance with the standard protocol recommended by the manufacturer on the product manual to generate mutant sequences of TCONS_00006091 containing the mutated binding sites of miR-153, miR-370 and let-7g, respectively, and the mutant sequences of TCONS_00006091 were also cloned into separate pcDNA luciferase vectors downstream of the luciferase report gene to generate mutant type plasmids of TCONS_00006091. Similarly, wild type and the mutant type sequences of SNAI1, IRS and HMGA2 containing the binding sites of miR-153, miR-370, and let-7g, respectively, were generated and cloned into pcDNA luciferase vectors downstream of the luciferase report gene to generate wild type and the mutant type plasmids of SNAI1, IRS and HMGA2 3’ UTR corresponding to miR-153, miR-370, and let-7g, respectively. In the next step, HSC-3 and SCC-9 cells were co-transfected with wild type or the mutant type plasmids of TCONS_00006091, SNAI1, IRS and HMGA2 in conjunction with their respective miRNAs. The transfection at 37 °C in a CO2 incubator for overnight using Lipofectamine 2000 (Invitrogen, Carlsbad, CA) in strict accordance with the standard protocol recommended by the transfection reagent manufacturer on the product manual. At 48 h post the transfection, the transfected cells were lysed by using a 1X lysis buffer before their luciferase activity was assessed on a luminometer (Berthold, Oak Ridge, TN) using a Dual Luciferase reporter gene assay kit (Promega, Madison, MI) in strict accordance with the standard protocol recommended by the assay kit manufacturer on the product manual.

### MTT assay

The logarithmic growth and viability of sample cells were evaluated by using an MTT assay kit (Thermo Fisher Scientific, Waltham, MA) in strict accordance with the standard protocol recommended by the manufacturer on the product manual.

### FCM assay

The apoptosis status of sample cells was evaluated by using flow cytometry (FACS Calibur, BD, San Jose, CA) and an Annexin V FITC-PI Dead Cell Apoptosis assay kit (Thermo Fisher Scientific, Waltham, MA) in strict accordance with the standard protocol recommended by the manufacturer on the product manual.

### Statistical analysis

All results were shown as mean ± SD. The differences between different groups were analyzed by utilizing Student’s tests and Prism 8.0 software (GraphPad, San Diego, CA). The difference was deemed significant when p < 0.05.

### Ethical approval

All experimental protocols were approved by the institutional ethics committee of Ningbo No.2 Hospital (Approval ID: SL-NBEY-KY-2022–148-01). Informed consent was obtained from all subjects or their legal guardians before the study. All methods were carried out in accordance with the latest version of Declaration of Helsinki.

## Results

### Patient recruitment and characteristics

The demographic and clinical characteristics of the two groups were observed and recorded in Table 1, which showed no obvious differences between the OSCC group and the Control group. Peripheral blood samples and oral mucosa tissue samples (cancerous and normal) were collected for subsequent analyses. Then, tissue samples from the OSCC group and the Control group were subjected to lncRNA microarray analyses, and 14 lncRNAs were identified (Supplementary Table 1) to exhibit evident changes (> twofold or < 0.5-fold).

**Table 1:** Demographic and clinical characteristics of patients with OSCC transformed from OLP (the OSCC group) and OLP with no sign of OSCC (the control group).

Characteristics	The control group (N = 102)	The OSCC group (N = 108)	*P* value
Age (years)			0.486
< 50	44	51	
≥ 50	58	57	
Sex			0.791
Male	63	63	
Female	39	45	
Smoking			0.568
Past or present	66	63	
Never	36	45	
Tumor location			
Tongue cancer		45	
Gingival carcinoma		18	
Carcinoma of the buccal Mucosa		24	
Others		21	
Tumor stage			
T1		25	
T2		48	
T3		25	
T4		10	

### The diagnostic value of TCONS_00006091, miR-153, miR-370 and let-7 in OSCC transformed from OLP

**Figure 1:**
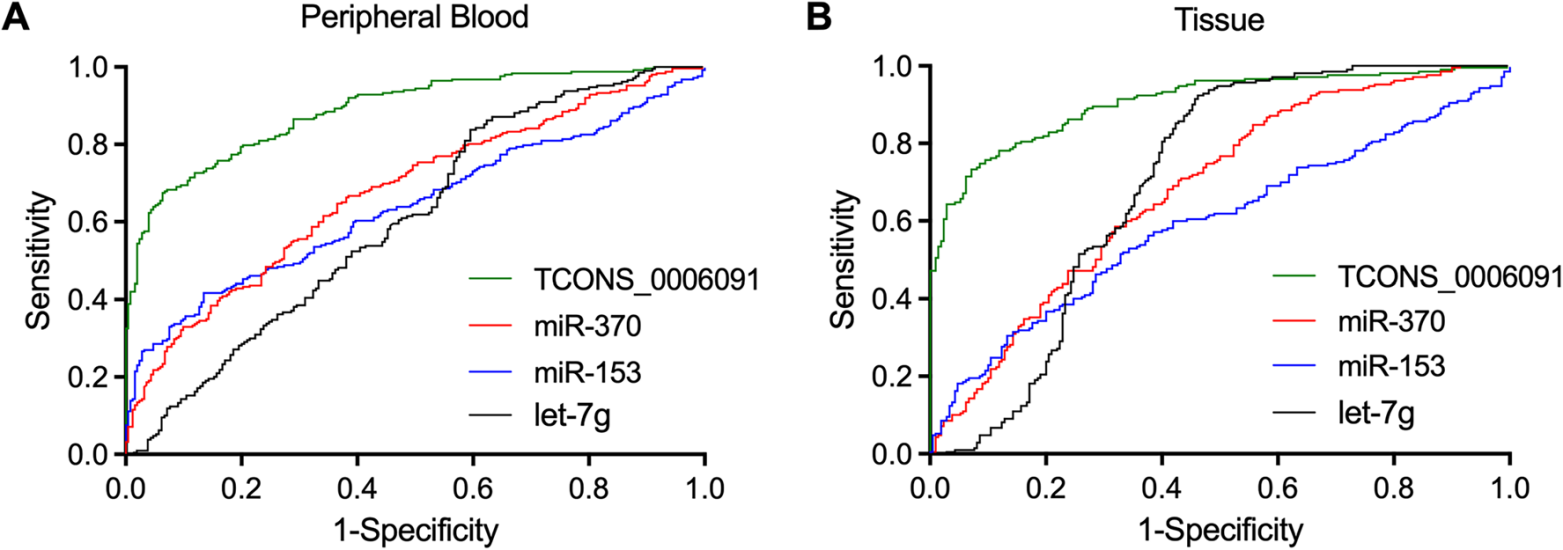
The values of TCONS_00006091, miR-153, miR-370 and let-7 in the diagnosis of malignant OLP. (**A**) ROC curves for TCONS_00006091, miR-153, miR-370, and let-7 in peripheral blood samples; (**B**) ROC curves for TCONS_00006091, miR-153, miR-370, and let-7 in tissue samples.

RT-qPCR was performed to observe the expression of TCONS_00006091, miR-153, miR-370, and let-7g in the peripheral blood samples collected from the OSCC group and the Control group. ROC curves were plotted, and AUC was calculated for TCONS_00006091, miR-153, miR-370, and let-7, respectively. As shown in Fig. 1A, the AUC of miR-153, miR-370 and let-7 was comparable, while the AUC of TCONS_00006091 was evidently higher. When repeating the above measurement and calculation on tissue samples (Fig. 1B), similar results were obtained. Therefore, it can be suggested that TCONS_00006091 exhibited a better value than miR-153, miR-370 and let-7 in the diagnosis of OSCC transformed from OLP.

### The expression of TCONS_00006091, miR-153, miR-370 and let-7g in the OSCC group and the control group

**Figure 2:**
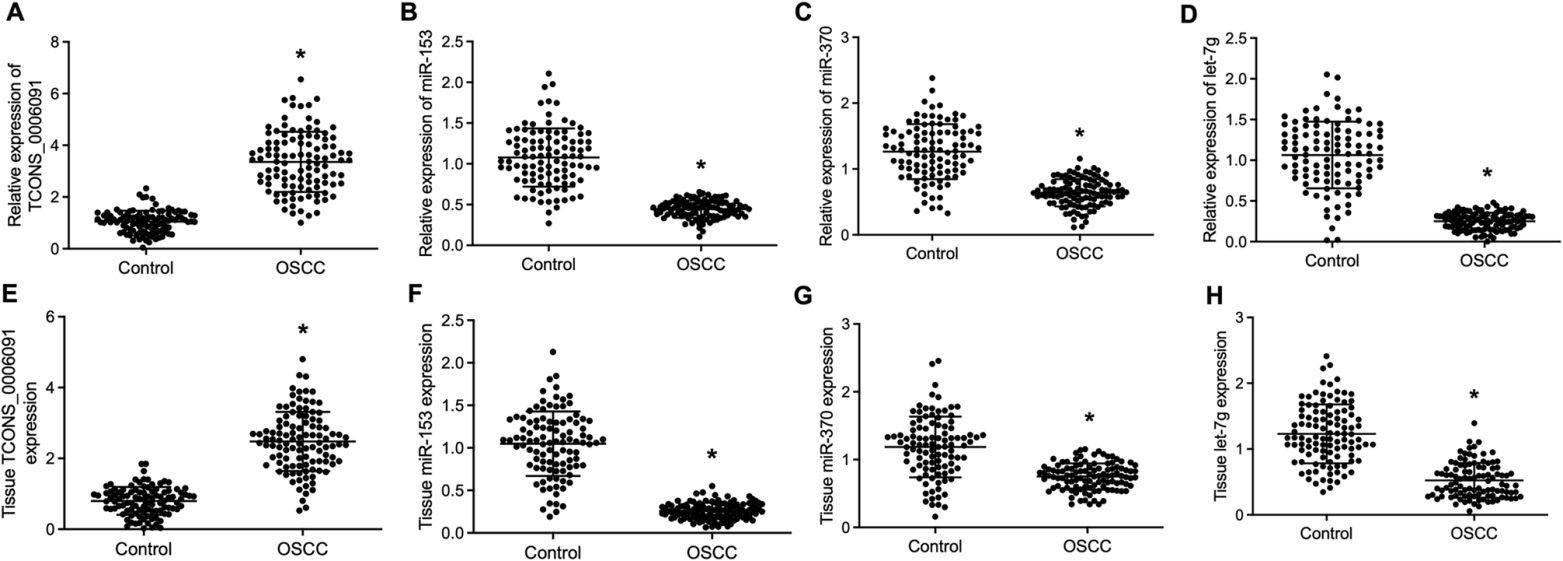
The expression of TCONS_00006091, miR-153, miR-370 and let-7g in the blood and tissue samples collected from the OSCC group and the Control group (* P value < 0.05 vs. the Control group). (**A**) Relative expression of blood TCONS_00006091 in the OSCC group and the Control group; (**B**) Relative expression of blood miR-153 in the OSCC group and the Control group; (**C**) Relative expression of blood miR-370 in the OSCC group and the Control group; (**D**) Relative expression of blood let-7g in the OSCC group and the Control group; (**E**) Relative expression of tissue TCONS_00006091 in the OSCC group and the Control group; (**F**) Relative expression of tissue miR-153 in the OSCC group and the Control group; (**G**) Relative expression of tissue miR-370 in the OSCC group and the Control group; (**H**) Relative expression of tissue let-7g in the OSCC group and the Control group.

RT-qPCR results of the peripheral blood samples collected from the OSCC group and the Control group showed increased TCONS_00006091 expression (Fig. 2A) in the OSCC group. The expression of miRNAs including miR-153 (Fig. 2B), miR-370 (Fig. 2C) and let-7g (Fig. 2D) was all significantly reduced in patients with OSCC transformed from OLP. Moreover, the above observation was also valid in tissue samples collected from the OSCC group and the Control group, which indicated evident up-regulation of TCONS_00006091 (Fig. 2E) and down-regulation of miRNAs including miR-153 (Fig. 2F), miR-370 (Fig. 2G) and let-7g (Fig. 2H).

### The mRNA and protein expressions of SNAI1, IRS and HMGA2 in the OSCC group and the control group

**Figure 3:**
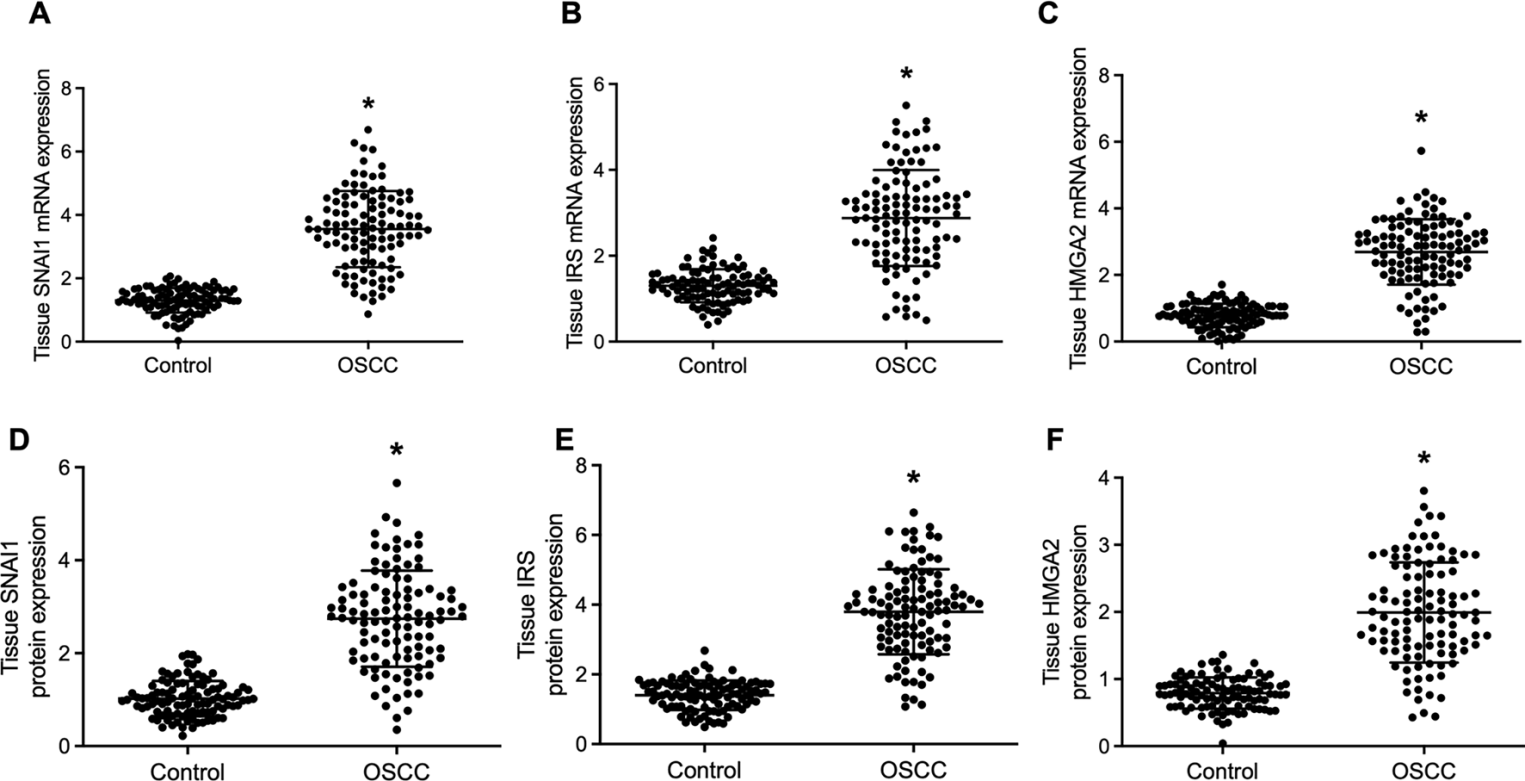
The expression of SNAI1 mRNA and protein, IRS mRNA and protein, and HMGA2 mRNA and protein in the tissues collected from the OSCC group and the Control group (* P value < 0.05 vs. the Control group). (**A**) Relative expression of tissue SNAI1 mRNA in the OSCC group and the Control group; (**B**) Relative expression of tissue IRS mRNA in the OSCC group and the Control group; (**C**) Relative expression of tissue HMGA2 mRNA in the OSCC group and the Control group; (**D**) Relative protein expression of SNAI1 in the OSCC group and the Control group; (**E**) Relative protein expression of IRS in the OSCC group and the Control group; (**F**) Relative protein expression of HMGA2 in the OSCC group and the Control group.

In the tissue samples, the mRNA and protein expressions of SNAI1, IRS and HMGA2 were measured. The RT-qPCR results showed significantly increased expression of SNAI1 mRNA (Fig. 3A), IRS mRNA (Fig. 3B) and HMGA2 mRNA (Fig. 3C) in patients with OSCC transformed from OLP. And the Western blot analysis presented markedly higher protein expression of SNAI1 (Fig. 3D), IRS (Fig. 3E) and HMGA2 (Fig. 3F) in patients with OSCC transformed from OLP.

### Establishment of TCONS_00006091 signaling pathways

**Figure 4:**
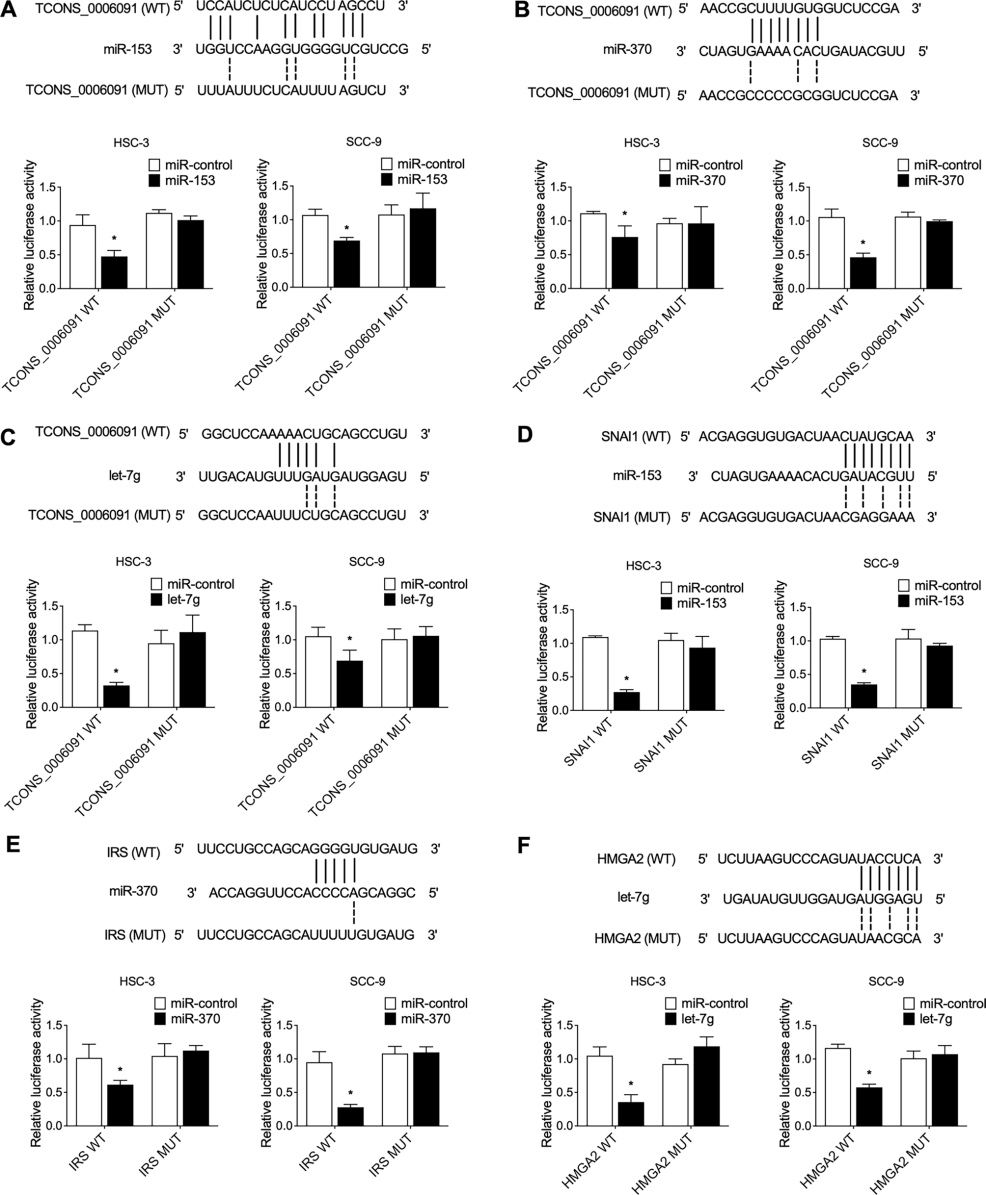
Establishment of TCONS_00006091 signaling pathways (*P value < 0.05 vs. miR control group). (**A**) Bioinformatic analysis and luciferase assay on the relationship between TCONS_00006091 and miR-153 in HSC-3 and SCC-9 cells; (**B**) Bioinformatic analysis and luciferase assay on the relationship between TCONS_00006091 and miR-370 in HSC-3 and SCC-9 cells; (**C**) Bioinformatic analysis and luciferase assay on the relationship between TCONS_00006091 and let-7g in HSC-3 and SCC-9 cells; (**D**) Bioinformatic analysis and luciferase assay on the relationship between miR-153 and SNAI1 mRNA in HSC-3 and SCC-9 cells; (**E**) Bioinformatic analysis and luciferase assay on the relationship between miR-370 and IRS mRNA in HSC-3 and SCC-9 cells; (**F**) Bioinformatic analysis and luciferase assay on the relationship between let-7g and HMGA2 in HSC-3 and SCC-9 cells.

Bioinformatic tools were utilized to predict the potential relationships between TCONS_00006091 and miR-153 (Fig. 4A), TCONS_00006091 and miR-370 (Fig. 4B), TCONS_00006091 and let-7g (Fig. 4C), miR-153 and SNAI1 mRNA (Fig. 4D), miR-370 and IRS mRNA (Fig. 4E), and let-7g and HMGA2 (Fig. 4F). Accordingly, putative binding sites were identified. Then, luciferase assays were performed in HSC-3 and SCC-9 cells to validate the above predictions. As indicated by the luciferase assay results, the relative luciferase activities of TCONS_00006091 in HSC-3 or SCC-9 cells were evidently reduced upon the transfection of miR-153 (Fig. 4A), miR-370 (Fig. 4B), and let-7g (Fig. 4C), validating that miR-153, miR-370 and let-7g were respectively sponged by TCONS_00006091. Moreover, the co-transfection of miR-153 and SNAI1 mRNA (Fig. 4D), miR-370 and IRS mRNA (Fig. 4E) or let-7g and HMGA2 (Fig. 4F) all inhibited the luciferase activities in HSC-3 or SCC-9 cells, indicating that SNAI1 mRNA, IRS mRNA and HMGA2 mRNA were respectively targeted by miR-153, miR-370 and let-7g, thus establishing the signaling pathways of TCONS_00006091/miR-153/SNAI1, TCONS_00006091/miR-370/IRS and TCONS_00006091/let-7g/HMGA2.

### Over-expression of TCONS_00006091 promoted cell proliferation and suppressed cell apoptosis

**Figure 5:**
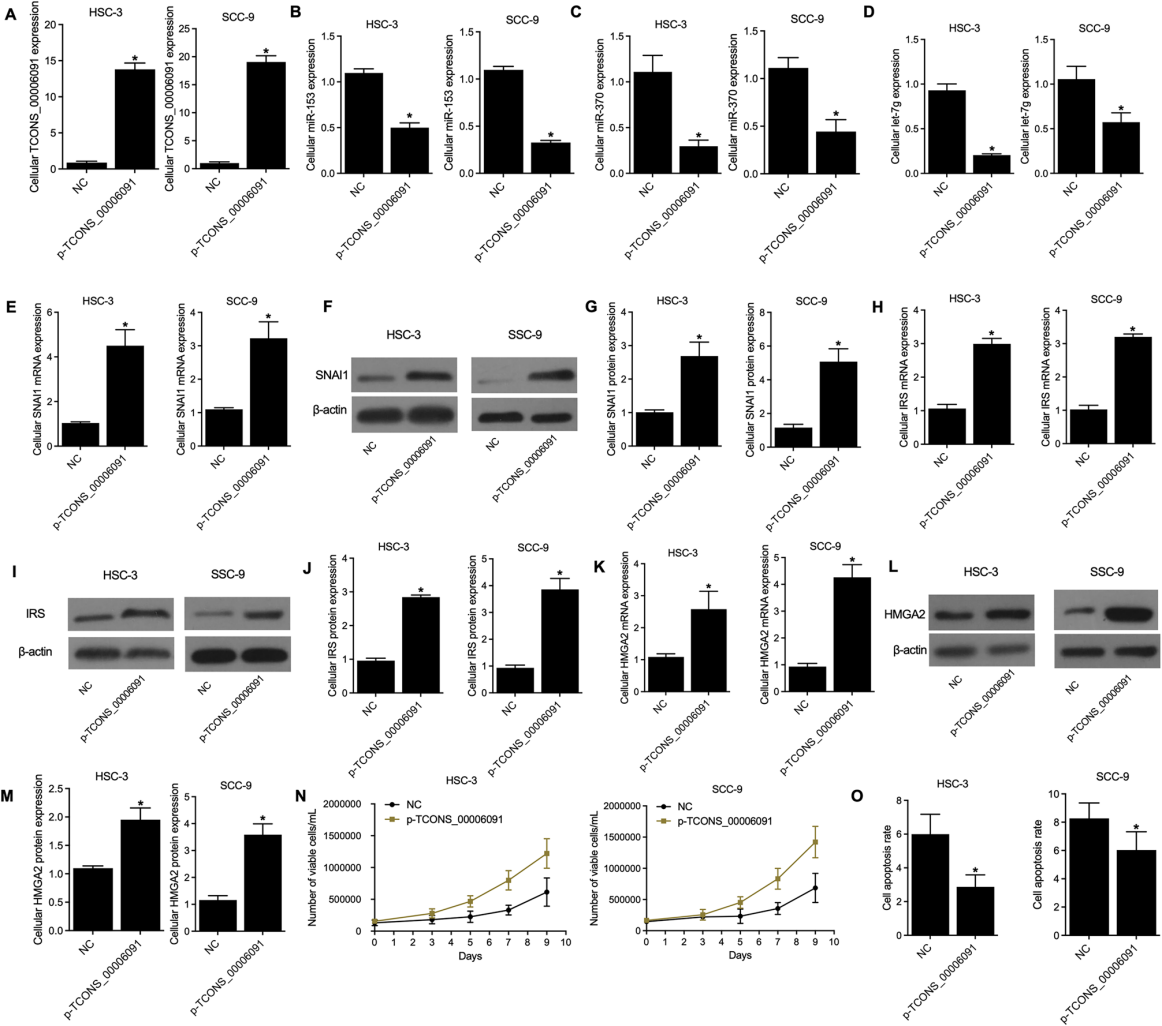
Over-expression of TCONS_00006091 regulated the gene and protein expressions, as well as the proliferation and apoptosis of HSC-3 and SCC-9 cells (* P value < 0.05 vs. NC group). (**A**) Relative expression of TCONS_00006091 in p-TCONS_00006091 and NC groups; (**B**) Relative expression of miR-153 in p-TCONS_00006091 and NC groups; (**C**) Relative expression of miR-370 in p-TCONS_00006091 and NC groups; (**D**) Relative expression of let-7g in p-TCONS_00006091 and NC groups; (**E**) Relative mRNA expression of SNAI1 in p-TCONS_00006091 and NC groups; (**F**) Western blot results of SNAI1 protein in p-TCONS_00006091 and NC groups; (**G**) Relative protein expression of SNAI1 in p-TCONS_00006091 and NC groups; (**H**) Relative mRNA expression of IRS in p-TCONS_00006091 and NC groups; (**I**) Western blot results of IRS protein in p-TCONS_00006091 and NC groups; (**J**) Relative protein expression of IRS in p-TCONS_00006091 and NC groups; (**K**) Relative mRNA expression of HMGA2 in p-TCONS_00006091 and NC groups; (**L**) Western blot results of HMGA2 protein in p-TCONS_00006091 and NC groups; (**M**) Relative protein expression of HMGA2 in p-TCONS_00006091 and NC groups; (**N**) The number of viable cells in p-TCONS_00006091 and NC groups detected by MTT assays; (**O**) The cell apoptosis rate in p-TCONS_00006091 and NC groups detected by FCM assays.

TCONS_00006091 was over-expressed in HSC-3 and SCC-9 cells transfected with plasmids carrying TCONS_00006091 (Fig. 5A). The over-expression of TCONS_00006091 also suppressed the expression of miRNAs including miR-153 (Fig. 5B), miR-370 (Fig. 5C) and let-7g (Fig. 5D) while increasing the expression of SNAI1 mRNA (Fig. 5E) and protein (Fig. 5F,G), IRS mRNA (Fig. 5H) and protein (Fig. 5I,J), and HMGA2 mRNA (Fig. 5K) and protein (Fig. 5L,M). Moreover, with the over-expression of TCONS_00006091, MTT assays showed that the number of viable cells (Fig. 5N) also showed an increasing trend, while the cell apoptosis rate (Fig. 5O) detected by FCM assays was reduced.

### Validation of the established TCONS_00006091 signaling pathways

**Figure 6:**
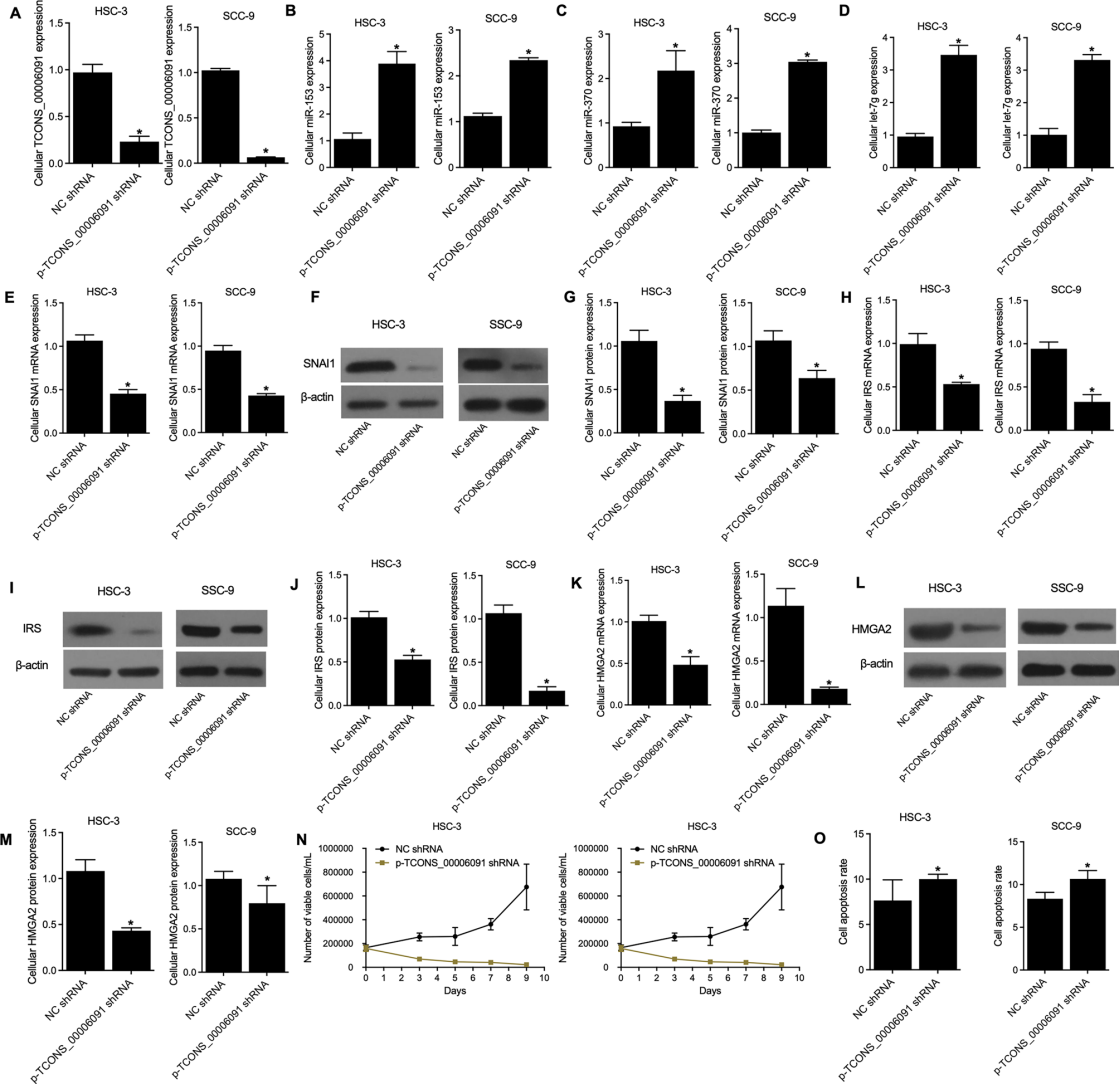
Knockdown of TCONS_00006091 regulated the gene and protein expressions, as well as the proliferation and apoptosis of HSC-3 and SCC-9 cells (* P value < 0.05 vs. NC group). (**A**) Relative expression of TCONS_00006091 in p-TCONS_00006091 shRNA and NC shRNA groups; (**B**) Relative expression of miR-153 in p-TCONS_00006091 shRNA and NC shRNA groups; (**C**) Relative expression of miR-370 in p-TCONS_00006091 shRNA and NC shRNA groups; (**D**) Relative expression of let-7g in p-TCONS_00006091 shRNA and NC shRNA groups; (**E**) Relative mRNA expression of SNAI1 in p-TCONS_00006091 shRNA and NC shRNA groups; (**F**) Western blot results of SNAI1 protein in p-TCONS_00006091 shRNA and NC shRNA groups; (**G**) Relative protein expression of SNAI1 in p-TCONS_00006091 shRNA and NC shRNA groups; (**H**) Relative mRNA expression of IRS in p-TCONS_00006091 shRNA and NC shRNA groups; (**I**) Western blot results of IRS protein in p-TCONS_00006091 shRNA and NC shRNA groups; (**J**) Relative protein expression of IRS in p-TCONS_00006091 shRNA and NC shRNA groups; (**K**) Relative mRNA expression of HMGA2 in p-TCONS_00006091 shRNA and NC shRNA groups; (**L**) Western blot results of HMGA2 protein in p-TCONS_00006091 shRNA and NC shRNA groups; (**M**) Relative protein expression of HMGA2 in p-TCONS_00006091 shRNA and NC shRNA groups; (**N**) The number of viable cells in p-TCONS_00006091 shRNA and NC shRNA groups detected by MTT assays; (**O**) The cell apoptosis rate in p-TCONS_00006091 shRNA and NC shRNA groups detected by FCM assays.

HSC-3 and SCC-9 cells were transfected with TCONS_00006091 shRNA to validate the established TCONS_00006091 signaling pathways. Accordingly, the knockdown of TCONS_00006091 (Fig. 6A) promoted the levels of miR-153 (Fig. 6B), miR-370 (Fig. 6C) and let-7g (Fig. 6D) while inhibited the levels of SNAI1 mRNA (Fig. 6E) and protein (Fig. 6F,G), IRS mRNA (Fig. 6H) and protein (Fig. 6I,J), and HMGA2 mRNA (Fig. 6K) and protein (Fig. 6L,M). Also, MTT assay results showed that the knockdown of TCONS_00006091 also inhibited cell viability (Fig. 6N) and FCM analysis showed that the knockdown of TCONS_00006091 up-regulated the apoptosis rate (Fig. 6O) of HSC-3 and SCC-9 cells.

## Discussion

The demographic and clinical characteristics of the OSCC group and the Control group had no obvious differences. In this study, we collected samples from patients with OSCC transformed from OLP (the OSCC group) and OLP with no sign of OSCC (the Control group), and performed lncRNA microarrays to identify the differentially expressed lncRNAs. The lncRNA microarray identified 14 lncRNAs which exhibited evident changes (fold-change > 2). Furthermore, we found that the OSCC group showed increased expression of TCONS_00006091 and decreased expression of miRNAs including miR-153, miR-370 and let-7g.

It has been demonstrated that SNAI1 acts as a miR-153 target. SNAI1 was also deemed as an essential factor promoting the metastasis of various cancers through inducing EMT^27^. On the other hand, it was revealed that miR-153, the targeting miRNA of SNAI1 and an independent marker for the prognosis of prostate cancer, suppressed cancer cell invasion by targeting the expression of SNAI1. It was also presented that the knockdown of AXL as well as β-catenin reduced SNAI1 expression. Several independent studies presented that the transcription factors with the ability to induce EMT can also enhance AXL expression to further upregulate the expression levels of Slug, Twist, as well as SNAI1 through positive feedback^28,29^. In another study, it was validated that AKT/GSK-3β/β-catenin signaling was responsible for mediating the progression of OSCC transformed from OLP via miR-34a-5p and AXL^30^.

Previous research revealed a negative correlation between the expression levels of IRS-1 and miR-370, confirming the theory that IRS-1 expression is targeted by miR-370 in OSCC^12^. The IRS protein belongs to the family of cytoplasmic adaptors with the ability to transmit signals generated from insulin as well as IGF-1 receptors to induce an intracellular response^31^. It was found that the expression level of IRS-2 was considerably upregulated in patients with OSCC transformed from OLP, while the silencing of IRS-2 expression suppressed cancerous cell proliferation through the Akt pathway^32^. The IRS-1 knockdown in SAS cells suppressed the migration as well as AIG of the cells while causing no inhibition in cell growth. Since the phenotypic effect of IRS-1 is mediated by miR-370, this result suggests that miR-370 plays a regulatory role in the progression of OSCC transformed from OLP via IRS-1^12^. In this study, bioinformatic tools and luciferase assays validated that miR-153, miR-370 and let-7g were respectively sponged by TCONS_00006091, while SNAI1 mRNA, IRS mRNA and HMGA2 mRNA was respectively targeted by miR-153, miR-370 and let-7g. The mRNA and protein expression of SNAI1, IRS and HMGA2 were all significantly increased in patients with OSCC transformed from OLP. In the in vitro analysis, we also found that the over-expression of TCONS_00006091 suppressed the expression of miR-153, miR-370 and let-7g while increasing the mRNA and protein expression of SNAI1, IRS and HMGA2. Also, cell proliferation was validated to be promoted by the over-expression of TCONS_00006091. In addition, the knockdown of TCONS_00006091 promoted the levels of miR-153, miR-370 and let-7g while inhibited the mRNA and protein levels of SNAI1 and IRS, leading to the inhibited cell proliferation and the promoted cell apoptosis.

A previous study used luciferase assays to show that HMGA2 was a direct target of let-7g-5p, and the presence of let-7g-5p downregulated the expression level of HMGA2. Other studies have shown that the family of miR-let-7 genes is closely related to HMGA2 expression. For example, let-7 was discovered to serve as a tumor suppressor via targeting the expression of HMGA2 in colon cancer^33^. HMGA2 was shown to be over-expressed during cancer progression by affecting a number of biological processes including the differentiation, survival, as well as apoptosis of cancer cells^34^. It was additionally suggested that in breast and colon cancers, HMGA2 induced the metastasis as well as invasion of cancer cells through TGFβ signaling^35^. On the other hand, numerous miRNAs target HMGA2 to exert their effects on many forms of cancers^36^. HGMA2 reduces the inhibitory effect of miR-124-3p on the migration, proliferation, as well as invasion of OSCC cells. Additionally, the positive staining of HGMA2 in tissues of oral cancer is related to various clinicopathological indicators such as the presence of cervical metastasis^37^. In this study, we also performed ROC analysis and found that TCONS_00006091 exhibited a better value than miR-153, miR-370 and let-7 in the diagnosis of OSCC transformed from OLP.

Nevertheless, our study's findings have limitations. A significant factor contributing to these limitations is the inclusion of patients who have histories of smoking or drinking habits, as both smoking and alcohol consumption are known to be potential catalysts for cancer development and progression.

## Conclusion

In this study, we demonstrated that TCONS_00006091 was up-regulated in patients with OSCC transformed from OLP to down-regulate the expression of miR-153, miR-370 and let-7g. These miRNAs were proved to respectively target the expression of SNAI1, IRS and HMGA2.

### Supplementary Information

Supplementary Information 1.

Supplementary Information 2.

## Data Availability

The datasets used and/or analysed during the current study available from the corresponding author on reasonable request.
