# Supplementary material for: Dysregulation of TCONS_00006091 contributes to the elevated risk of oral squamous cell carcinoma by upregulating SNAI1, IRS and HMGA2
Source: Sci Rep. 2024 Apr 26;14:9616. doi: 10.1038/s41598-024-60310-4 (PMC11053020; doi:10.1038/s41598-024-60310-4)

beta actin/HSC-3 for Fig 5F

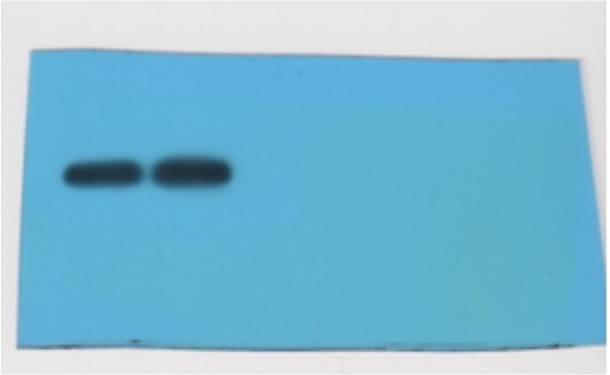

beta actin/HSC-3 for Fig 5I

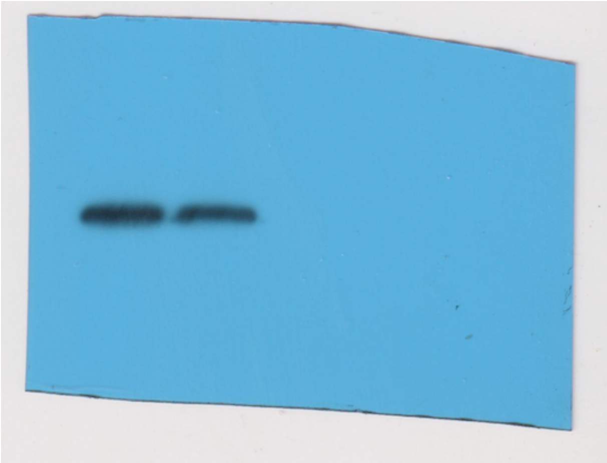

beta actin/HSC-3 for Fig 5L

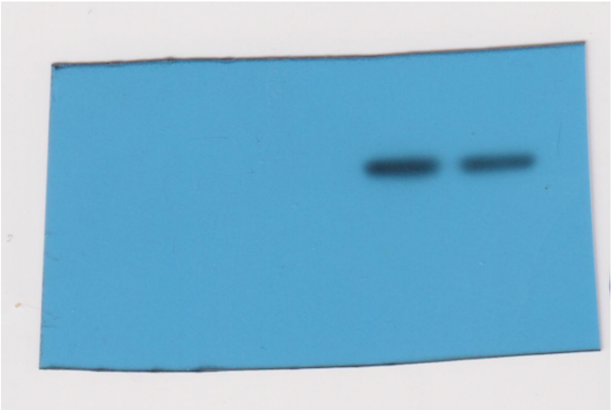

beta actin/HSC-3 for Fig 6F

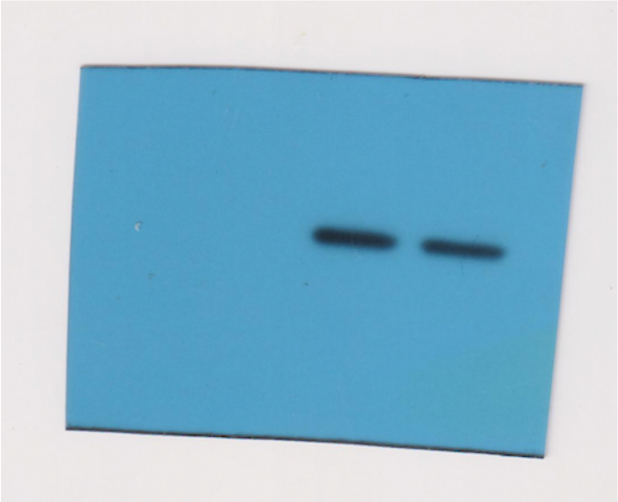

beta actin/HSC-3 for Fig 6I

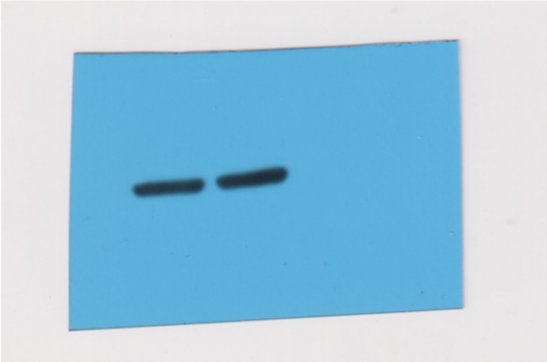

beta actin/HSC-3 for Fig 6L

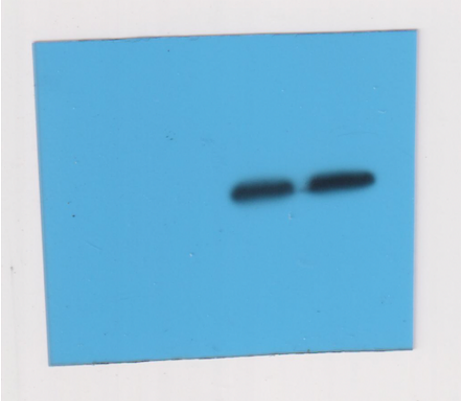

beta actin/SSC-9 for Fig 5F

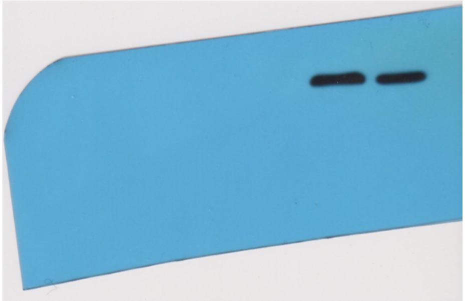

beta actin/SSC-9 for Fig 5I

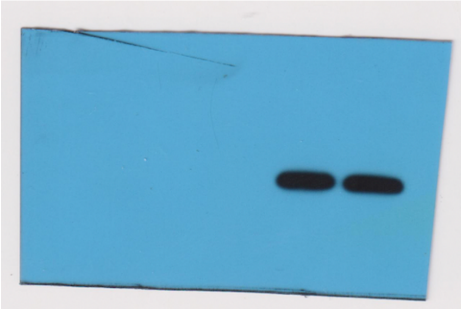

beta actin/SSC-9 for Fig 5L

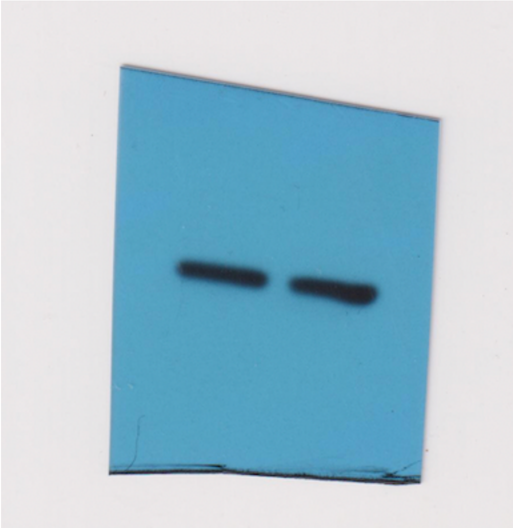

beta actin/SSC-9 for Fig 6F

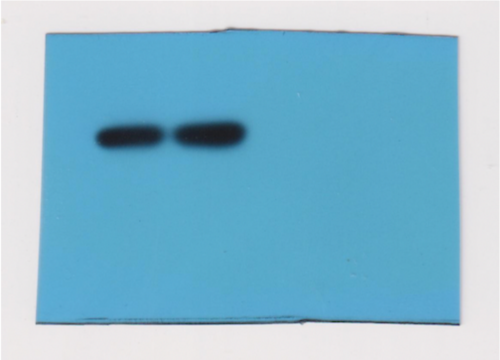

beta actin/SSC-9 for Fig 6I

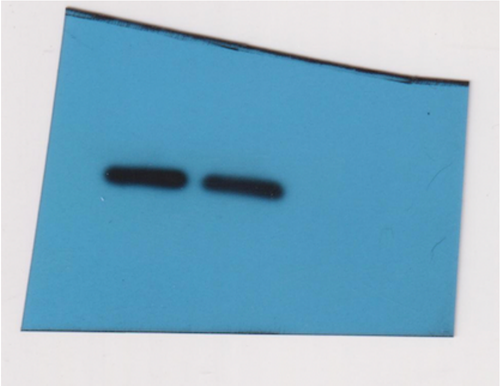

beta-actin/SSC-9 for Fig 6L

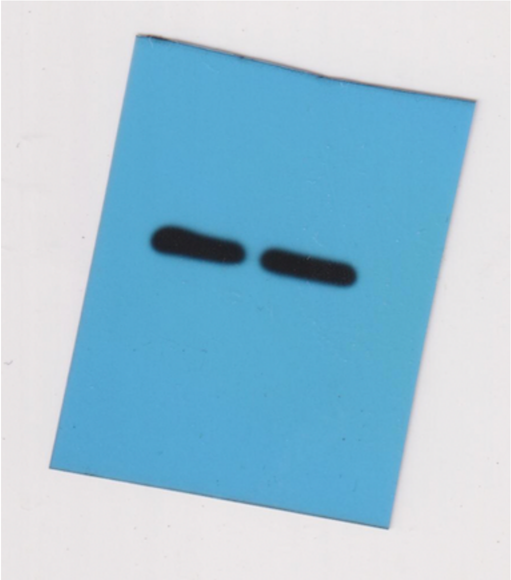

HMGA2/HSC-3 for Fig 5L

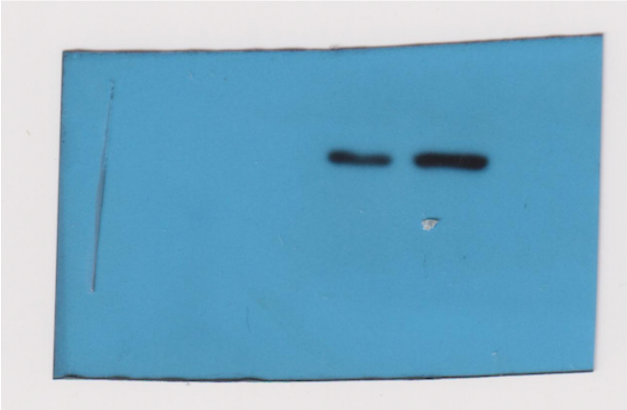

HMGA2/HSC-3 for Fig 6L

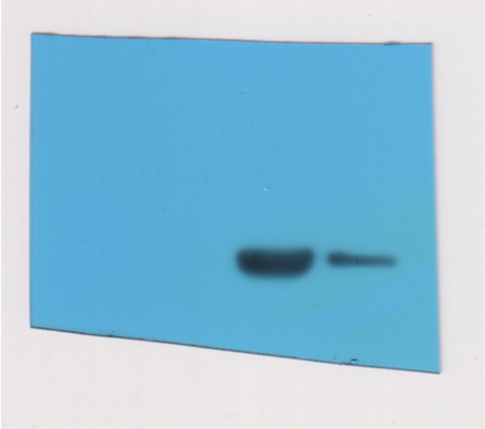

HMGA2/SSC-9 for Fig 5L

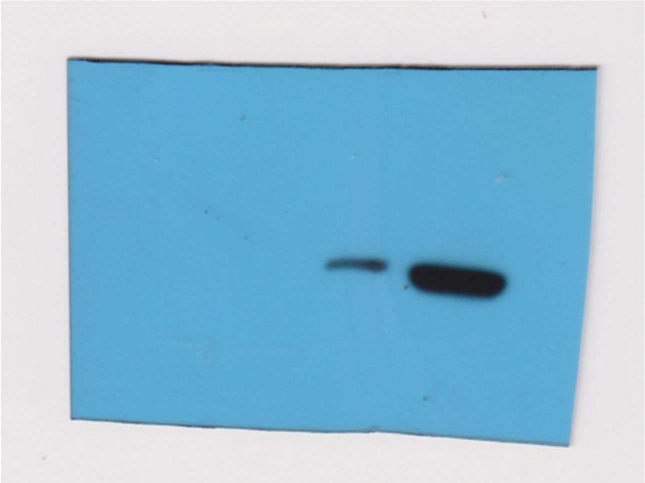

HMGA2/SSC-9 for Fig 6L

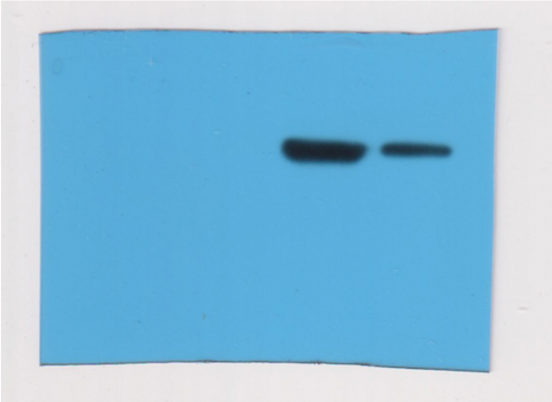

IRS/HSC-3 for Fig 5I

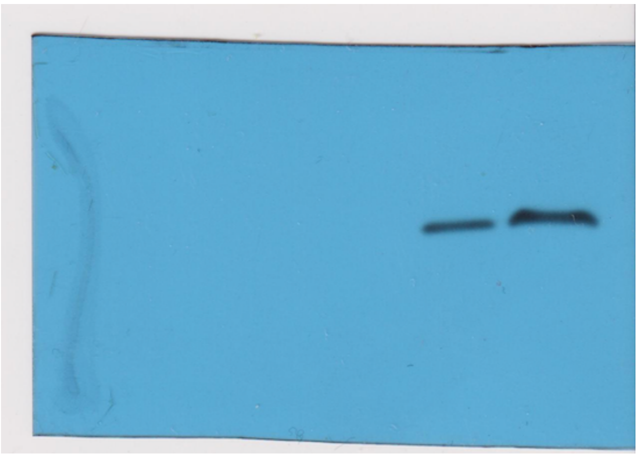

IRS/HSC-3 for Fig 6I

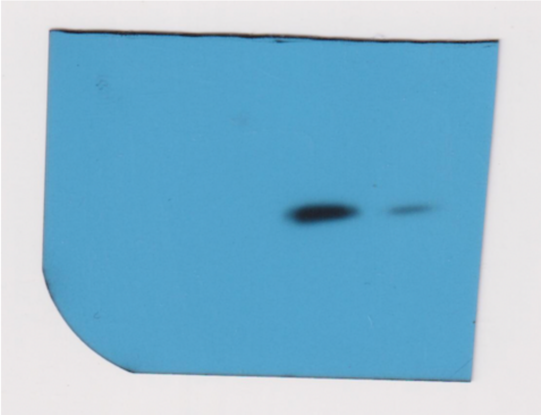

IRS/SSC-9 for Fig 5I

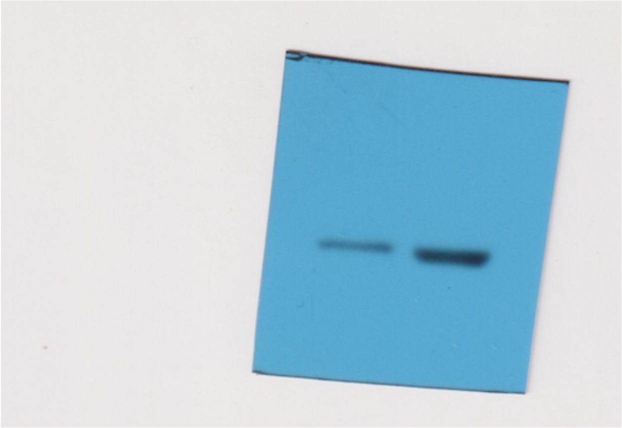

IRS/SSC-9 for Fig 6I

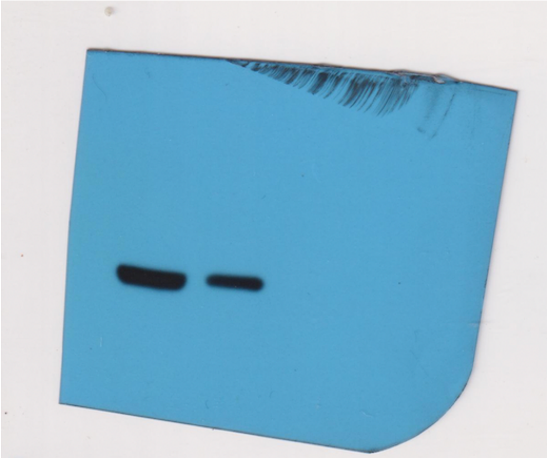

SNAI1/HSC-3 for Fig 5F

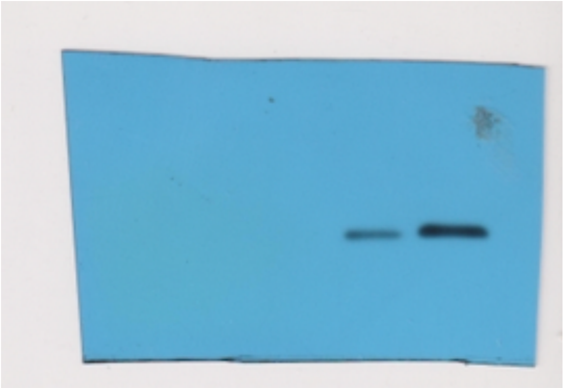

SNAI1/HSC-3 for Fig 6F

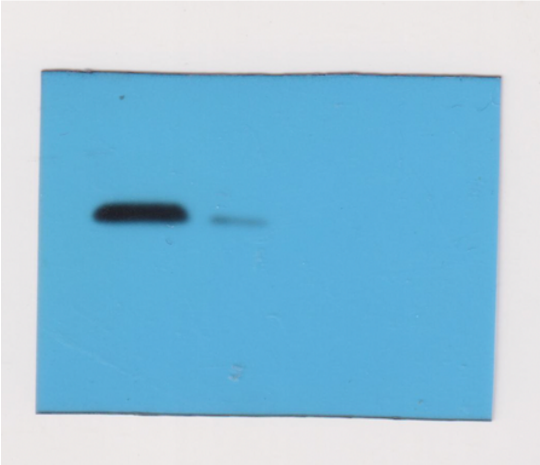

SNAI1/SSC-9 for Fig 5F

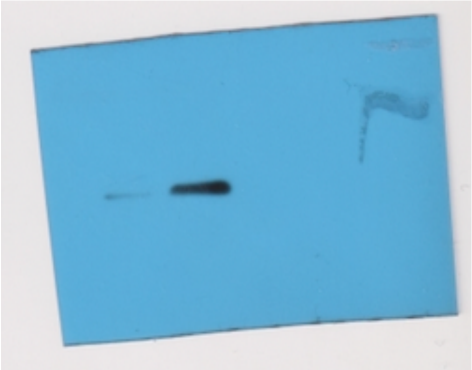

SNAI1/SSC-9 for Fig 6F

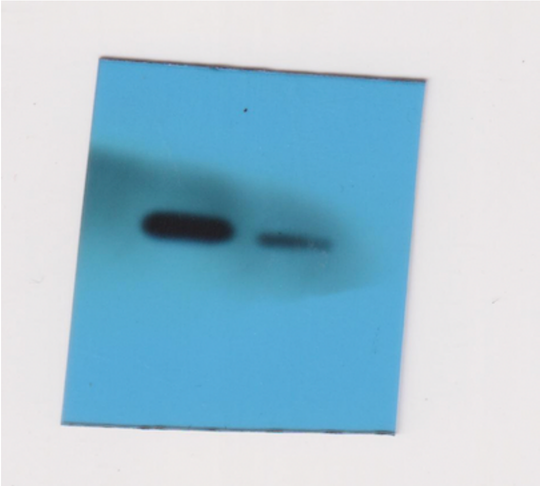

Supplement: Supplementary file 1 — Supplementary Information 1. [file 41598_2024_60310_MOESM1_ESM.pdf]
