# Supplementary material for: Dysregulation of TCONS_00006091 contributes to the elevated risk of oral squamous cell carcinoma by upregulating SNAI1, IRS and HMGA2
Source: Sci Rep. 2024 Apr 26;14:9616. doi: 10.1038/s41598-024-60310-4 (PMC11053020; doi:10.1038/s41598-024-60310-4)
Supplement: Supplementary file 2 — Supplementary Information 2. [file 41598_2024_60310_MOESM2_ESM.docx]

| Supplementary Table 1. List of lncRNAs with evident changes in the lncRNA microarray analysis | | | |
| --- | --- | --- | --- |
| **lncRNA** | **The control group** | **The OSCC group** | **Fold change** |
| **Up-regulated lncRNA** |  |  |  |
| TCONS_00090092_MEG3 | 0.458 | 1.854 | 4.048 |
| TCONS_I2_00006843 | 0.115 | 0.358 | 3.113 |
| TCONS_I2_00000179 | 2.872 | 8.147 | 3.834 |
| TCONS_I2_00014091 | 0.884 | 3.387 | 3.831 |
| TCONS_I2_00004424 | 0.167 | 0.687 | 4.114 |
| TCONS_00006091 | 1.358 | 3.475 | 2.559 |
| lnc‑CXCR3‑5:2 | 0.2884 | 2.218 | 7.691 |
| ENST00000433410 | 1.188 | 6.352 | 5.347 |
|  |  |  |  |
| **Down-regulated lncRNA** |  |  |  |
| TCONS_I2_00018070 | 0.684 | 0.084 | 0.123 |
| TCONS_I2_00018071 | 0.548 | 0.147 | 0.268 |
| TCONS_I2_00021262 | 0.879 | 0.318 | 0.362 |
| TCONS_I2_00030560 | 4.584 | 1.358 | 0.296 |
| lnc‑KRT79‑2:1 | 2.658 | 0.875 | 0.329 |
| ENST00000450445 | 0.448 | 0.105 | 0.234 |
